# Neutropenia and Felty Syndrome in the Twenty-First Century: Redefining Ancient Concepts in Rheumatoid Arthritis Patients

**DOI:** 10.3390/jcm13247677

**Published:** 2024-12-17

**Authors:** Jorge Luis Rodas Flores, Blanca Hernández-Cruz, Víctor Sánchez-Margalet, Ana Fernández-Reboul Fernández, Esther Fernández Panadero, Gracia Moral García, José Javier Pérez Venegas

**Affiliations:** 1 Rheumatology Department, Virgen Macarena University Hospital, Health Service of Andalucian, 41009 Seville, Spain; jorgel.rodas.sspa@juntadeandalucia.es (J.L.R.F.); ana.fernandez.sspa@juntadeandalucia.es (A.F.-R.F.); esther.fernandez.panadero.sspa@juntadeandalucia.es (E.F.P.); gracia.moral.sspa@juntadeandalucia.es (G.M.G.); jjavier.perez.sspa@juntadeandalucia.es (J.J.P.V.); 2 Department of Medical Biochemistry and Molecular Biology and Immunology, School of Medicine, Virgen Macarena University Hospital, 41009 Seville, Spain; margalet@us.es

**Keywords:** rheumatoid arthritis, neutropenia, DMARDs, Felty syndrome

## Abstract

**Objectives:** To describe the frequency of neutropenia and Felty syndrome in patients with rheumatoid arthritis (RA) attended in routine clinical practice. **Methods:** We selected by randomization a sample of 270 RA patients attended from January 2014 to November 2022. Demographic, clinical, and neutropenia-related variables were collected from the electronic medical records. Neutropenia was defined as having an absolute neutrophil count (ANC) of less than 1500/mm^3^ once, and acute if it persisted for <3 months. Felty syndrome was defined as RA-related neutropenia, rheumatoid factor (RF) and/or anti citrullinated protein antibody (ACPA) positivity. **Results:** We found 50 patients who had at least one neutropenia episode, with an incidence of 18.5% (14.0–25.6%). Most were women, with age (mean, p25–p75) at the time of neutropenia of 61.5 (57.4–69.3) years, 85% RF+ and 76% ACPA+. The demographic and RA characteristics of patients with and without neutropenia were very similar, except for sex: most patients with neutropenia were women. The 50 patients had 99 episodes of neutropenia; 59% were acute. The lower ANC was 1240 (1000–1395) mm^3^, and most of the episodes were mild (74%). In 32% of cases, there was other cytopenia. The RA activity measured by DAS28 in patients with neutropenia was low, at 2.18 (1.75–2.97). A total of 82 of 99 neutropenia episodes were related to DMARDs, 60% to Anti-IL6 drugs in monotherapy, 13% to RA activity, 3% to infectious diseases and 1% to hematologic malignancy. There were five (1.8%) cases with Felty syndrome, but only one woman with the classic combination of RA, positivity of autoantibodies (RF and ACPA), neutropenia and splenomegaly. **Conclusions:** In the 21st century, neutropenia in RA patients is most commonly related to biologics, mostly IL6 inhibitors and methotrexate. Episodes are mild, acute, with low RA activity, and associated with severe infections in few cases. Felty syndrome is rare.

## 1. Introduction

Neutropenia in patients with rheumatoid arthritis (RA) occurs in 0.5 to 37% of cases depending on several variables, mainly involving treatment with disease-modifying anti-rheumatic drugs (DMARDs) [1,2,3,4]. In patients with early untreated RA, the frequency of neutropenia is as low as 0.65–1.4% [3,4]. For RA patients treated with synthetic conventional DMARDs (scDMARDs), this rate is about 8% (2–12%) [1]. In those treated with biologics (bDMARDs), it is 14–19%, with higher values for IL6-inhibitors (IL6i) like tocilizumab (1–10%) and anti-CD20 drugs like rituximab (3–27%) and lower ones for tumoral necrosis factor inhibitors (TNFi) (2–3%) [1,2]. In those treated with Janus Kinase inhibitors (JAKi), the rate is about 5% [1,2]. A recent study estimated the frequency of neutropenia in early AR, finding a value of 7.5% [1]. Neutropenia increases with the combination of scDMARDs and bDMARDs, and significantly increases comorbidity and complicates the management of the disease with DMARDs within the treat-to-target strategy [3,4]. The most common complication of neutropenia, whatever the cause, is infection, which can be serious [1,2,3]. The risk of severe infection is greater if neutropenia is severe, if it appears quickly and with a longer duration, if it is associated with chronic foci of infections, previous infections, anemia or other cytopenia and if it occurs in the presence of certain comorbidities (chronic kidney disease, lung pathology, heart failure or chronic infections). And the risk is mainly for bacterial infections [1,2,3]. 

The etiology of neutropenia in RA patients is complex. Neutropenia can be congenital, a very rare condition that occurs with the same frequency as in individuals without RA, or acquired, which is the most common. The causes of acquired neutropenia are i. peripheral destruction by an autoimmune reaction or toxics (drugs as DMARDs or others); ii. sequestration, either splenic (hypersplenism), in reticuloendothelial tissue or excess of vascular marginalization of neutrophils; and iii. inadequate neutrophil production in hematopoietic organs due to vitamin deficiency (i.e., folic acid deficiency in patients treated with methotrexate), tumor invasion or toxicity (drugs as DMARDS or others) [1,2,3,4].

A special form of RA was described in 1924 as a combination of RA, leucopenia and splenomegaly by Augustus Felty [5]. Currently, Felty syndrome is defined as the presence of neutropenia (absolute neutrophil counts of less than 1500× mm^3^), splenomegaly and typical antibodies (RF and/or ACPA) in RA patients. Although it has been described in association with other autoimmune conditions as systemic lupus erythematosus; in these cases, the term autoimmune neutropenia is preferred. Its prevalence is low and decreasing: 1% in 1985 to 0.5% in the 21st century [6]. Other common signs are extra-articular RA manifestations, fever, anemia, mucosal and skin ulcers, respiratory tract infections, thrombocytopenia and lymphadenopathy. These patients have poor prognostics [5,6].

The treatment of neutropenia depends on its etiology. If related to toxics, withdrawal of the drug or toxic substance or material is recommended. In some cases, the addition of folic acid supplementation is pursued. Optimizing the treatment of RA is useful, as in Felty syndrome. Neoplasia should be looked for and treated, for example, by the screening and treatment of large granular lymphocyte leukemia. In addition, the correct prevention of infections with vaccination or antibiotic prophylaxis must be pursued in specific cases, as well as the early treatment of associated infections [1,2,3].

The objectives of our study were to determine the frequency of neutropenia and Felty syndrome, and to determine its clinical characteristics.

## 2. Materials and Methods

An observational, retrospective and analytical study was designed. From a database of 858 patients with adult RA diagnosed according to ACR/EULAR criteria [7] treated in usual clinical practice in the Rheumatology Department of the Virgen Macarena University Hospital (a tertiary level hospital belonging to the Public Health System of Andalusia, in Seville, Spain) from January 2014 to November 2022, we selected a sample. The sample size was calculated based on an estimated prevalence of neutropenia of 10%, and the patients were selected by randomization with a computer program. Patients without an accurate RA diagnosis, those under 18 years of age, those with overlapping syndromes and those with other immune mediated diseases were excluded. Demographic, clinical, laboratory and neutropenia-specific data were collected from electronic medical records, with emphasis on the identification of neutropenia. Neutropenia information was cross-checked with electronic laboratory records. Data on treatment were reviewed in the electronic prescription, and its relationship with neutropenia was defined by the treating rheumatologist.

Definitions: Neutropenia was when the absolute neutrophil count (ANC) was ≤1500/mm^3^ in a determination. Mild neutropenia (grade II) was diagnosed in patients with an ANC of 1500 to 1000/mm^3^; moderate (grade III) of 1000 to 500/mm^3^; and severe (grade IV) < 500/mm^3^. Neutropenia was considered acute if it persisted <3 months and chronic if it persisted ≥3 months. Anemia was defined as having a hemoglobin level <13 g/dL (men) and <12 g/dL (women). Lymphopenia was defined as having an absolute lymphocyte count of <1000/mm^3^. Thrombocytopenia was defined as having an absolute platelet count of <140,000/mm^3^ (men) and <130,000/mm^3^ (women) [8,9]. Infection associated with the neutropenia process was that present within 3 months of the detected neutropenia episode. Felty syndrome was diagnosed if patients had rheumatoid factor positivity (RF+) and/or anti-citrullinated protein antibody positivity (ACPA+) and/or neutropenia attributed to RA activity; and classic Felty syndrome was if splenomegaly plus typical signs and symptoms were present.

Statistical analysis was performed by descriptive statistics with calculation of median and percentiles 25 and 75, percentages, and 95% confidence intervals. Nonparametric tests were used to calculate p values (Xi^2^ and Mann–Whitney U tests), assuming non-normal distribution of variables and unequal sample sizes. A logistic regression was performed to determinate the variables related to neutropenia. In them, the dependent variable was neutropenia, and the independent variables were those with clinical relevance and those that, in the univariate analysis, showed a *p* value ≤ 0.2. Analyses were performed using Stata version 13.1 (StataCorp LP, College Station, TX, USA). The study was approved by the regional Ethics and Clinical Research Committee of the Andalusian Health Service.

## 3. Results

Out of a base of 858 patients with RA who attended usual clinical practice, 270 (30%) were randomly selected. The sample included 213 (79%) women. The median age (percentile 25–percentile 75) at the time of the study was 61.5 (53.0–69.3) years. They had an RA of 10.5 (6.6–18.2) years; 84% had RF+, 78% ACPA+ and 72% both FR+ and ACPA+.

Of the 270 patients included, 50 (18.5% CI95% 14.0–25.6%) had at least one episode of neutropenia. The demographic and RA characteristics of both groups, with and without neutropenia, were very similar, except for sex, since most patients with neutropenia were women. Patients with neutropenia had other cytopenias, mainly thrombocytopenia. They used bDMARDs most frequently, without statistical differences, as shown in Table 1.

### 3.1. Multivariate Analysis

In the best logistic regression model, the variables that were associated with neutropenia were treatment with IL6i, the presence of other cytopenias, and female sex. Neither activity according DAS28, age or the duration of the RA showed association. The data are shown in Table 2.

### 3.2. Neutropenia Episodes Features

A total of 50 patients had 99 episodes of neutropenia (Table 3); 59% episodes were acute and 41% chronic. The lower ANC was 1240 (1000–1395) mm^3^, and most of the episodes were mild (74%). In 32%, there was other cytopenia: anemia in 19%, thrombocytopenia in 14% and lymphopenia in 9%.

Neutropenic episodes occurred in older patients with long-standing illnesses. Interestingly, the RA activity measured by DAS28 at each neutropenia episode was low, at 2.18 (1.75–2.97). The most common adverse event related to neutropenia was infection in 12 (12%) cases. Most infections were mild, auto-limited and resolved with symptomatic or oral antibiotic therapy; 6% of those were of the upper respiratory tract and 4% of the lower urinary tract. There were two (2%) severe infections: one case of herpes simplex keratitis and another of pneumonia by *K. pneumoniae*, which we describe below. 

Regarding etiology, neutropenia drugs (78%) and RA activity (13%) were the most common causes. Biologics were related to 79 episodes of neutropenia: IL6i in 54, with tocilizumab either in monotherapy (40%) or in combination with scDMARDs (19%) as the bDMARD. The second one was TNFi, in 24 episodes, etanercept either in monotherapy (11%) or combination (6%), followed by adalimumab and infliximab. The third was rituximab, with only one case. scDMARDs were, as a group, the second most common cause of neutropenia either in monotherapy or combination with biologics in 44 episodes. Methotrexate in monotherapy caused 20% of episodes, and methotrexate with bDMARDs caused 41% of episodes. The JAKi were related to neutropenia episodes only in 3% of cases. In addition, the treatment of oncologic conditions was related to two episodes (Table 4). In total, 86% of neutropenia episodes were resolved: 59% spontaneously, 31% with reduction in the dose of DMARD, 11% with stopping DMARD, and 4% changing DMARD.

#### 3.2.1. Description of Cases with Severe Infections

- Case 1. The patient is a 79-year-old woman with RA FR+, ACPA+, erosive and sicca symptoms with a duration of 23 years. She had received multiple scDMARDs and, since 2010, tocilizumab monotherapy, maintaining remission with DAS < 2.4. Her comorbidities include arterial hypertension and stable stage 3a chronic kidney disease. In October 2022, when she was receiving tocilizumab at a reduced dose (162 mg/2 weeks) during an episode of mild acute leucopenia and neutropenia for two months (total leukocytes 3990× mm^3^, total neutrophils 1310× mm^3^, total lymphocytes 1380× mm^3^, platelets 190,000× mm^3^, serum creatinine 1.02 mg/dL, glomerular filtration rate 53 mL/min, erythrocyte sedimentation rate (ESR) 7 mm/hour, C-Reactive protein (CRP) 0.3 mg/L), she was admitted to the emergency room with a red left eye, photophobia plus epiphora. The ophthalmologists established the diagnosis of herpetic keratitis, and she was treated on an outpatient basis with topical ganciclovir with resolution after 7 days, without recurrence. Tocilizumab was discontinued during the infection and restarted in reduced doses, according to the label [10]. She currently remains in remission with tocilizumab (162 mg/2 weeks/SC) and has not had infections, keratitis or neutropenia.
- Case 2. The patient is a 79-year-old woman with RA, FR+ ACPA+, erosive plus fibromyalgia and bronchial asthma of 25 years. After multiple scDMARDs, she started her first biologic, tocilizumab, in 2009, and maintained remission with reduced dose (4 mg/kg/IV each 28 days). The reduction in dose was due to good clinical response but moderate neutropenia. She was admitted due to fever, chills, cough with hemoptoic sputum plus left pleural pain during 5 days. A pulmonary condensation image in the left base and a positive blood culture for *Klebsiella pneumoniae* were confirmed. At the time of pneumonia, RA was in remission (DAS28VSG 2.1) without leukopenia or neutropenia (total leucocytes 6900× mm^3^, and total neutrophils 6430× mm^3^) but severe lymphopenia (130× mm^3^) and mild thrombocytopenia (total platelets 129,000× mm^3^), ESR 2 mm/hour, CRP 46.1 mg/L. After 10 days of IV antibiotic therapy, the condition resolved. Tocilizumab was restarted one month later and was discontinued after 10 months due to chronic moderate neutropenia, according to the label [10]. She was switched to etanercept, which is maintained to date at 50 mg/week SC. She is in remission, without infections and without neutropenia.

#### 3.2.2. Classic Felty Syndrome

Five of fifty (1.8%) patients with neutropenia had characteristics of Felty Syndrome, i.e., neutropenia plus RF+ and/or ACPA+, but just one of the three classic features (RA, FR+ and ACPA+, neutropenia and splenomegaly), for a prevalence of 0.3% of the total of patients.

**Figure 1:**
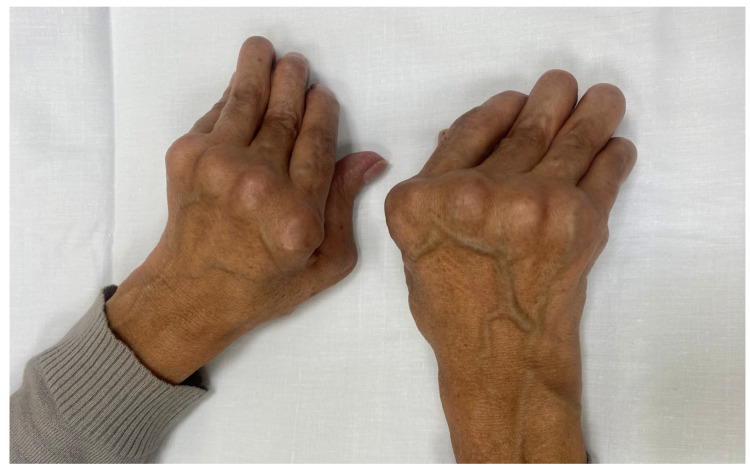
Hands of patient with Felty syndrome. She has characteristics swan neck deformity, metacarpophalangeal subluxation and ulnar blunt.

- Case 3. Felty Syndrome. The patient is a 46-year-old woman with RA according to EULAR/ACR criteria [7], with poor prognostic characteristics (high levels of activity, RF+, ACPA+, erosions and poor physical function) as evaluated in 2011, with poor compliance. From 2011 to 2015, she received irregular treatment with methotrexate, leflunomide, and low doses of oral prednisone and refused bDMARDs. In 2019, she returned to the clinic with six swollen joints, eight tender joints, 6 cm of visual analogue scale of pain (0 = no pain to 10 = maximum pain), and 6 cm of VAS of RA activity (0 = no activity to 10 = maximum activity), with DAS28PCR 5.07, HAQ 1.5 and poor perception of health. We found hand deformity with ulnar burst and swan neck fingers (Figure 1).

**Figure 2:**
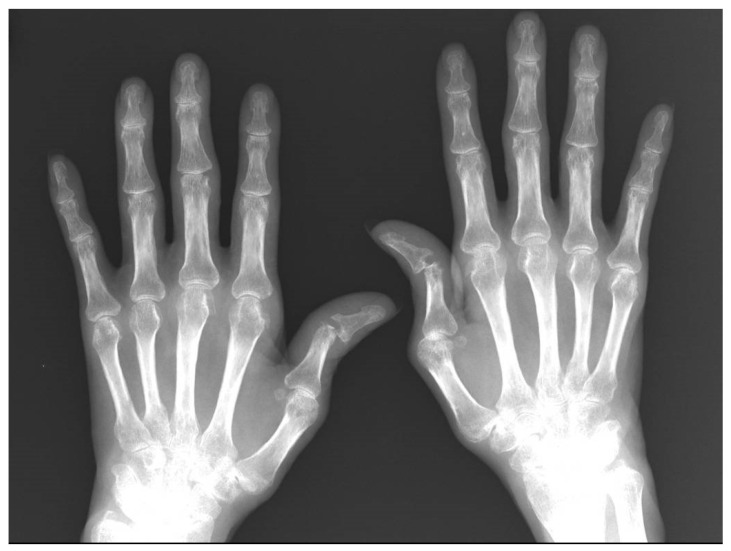
Anteroposterior hand X-ray with subluxation of the metacarpophalangeal and interphalangeal joints of first fingers, and erosions in metacarpal heads and proximal interphalangeal heads.

Her laboratory data were as follows: hemoglobin 10.7 g/dL, total leukocytes 3850× mm^3^, total neutrophils 1000× mm^3^, serum IgA 693 mg/dL, serum IgG 2706 mg/dL, serum IgM 868 mg/dL, FR 522 UI/dL, ACPA > 340 U/mL, ESR 35 mm/hora, CRP 15 mg/dL. Her X-rays showed ulnar subluxation of bilateral first finger interphalangeal, with erosions in carpals, metacarpophalangeal and proximal interphalangeal (Figure 2) as well as erosions in metatarsal heads. 

**Figure 3:**
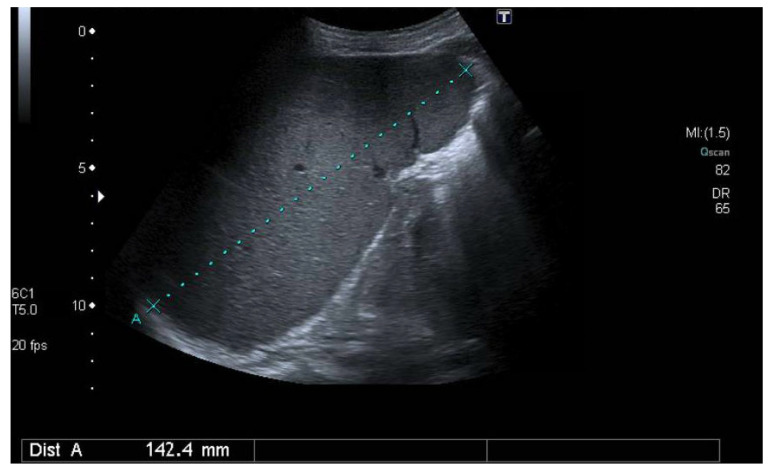
Splenomegaly with spleen of 14 cm.

Treatment was attempted with adalimumab and etanercept, which she did not tolerate and preferred low doses of prednisone (1.25 to 5 mg/day). She evolved poorly with persistent RA activity (DAS28-PCR index between 4.5 to 6.0) and pancytopenia (mild to moderate anemia, neutropenia, lymphopenia, and thrombocytopenia), without clinical manifestations. Abdominal ultrasound was performed for suspected Felty syndrome, confirming 14 cm splenomegaly (Figure 3). 

**Figure 4:**
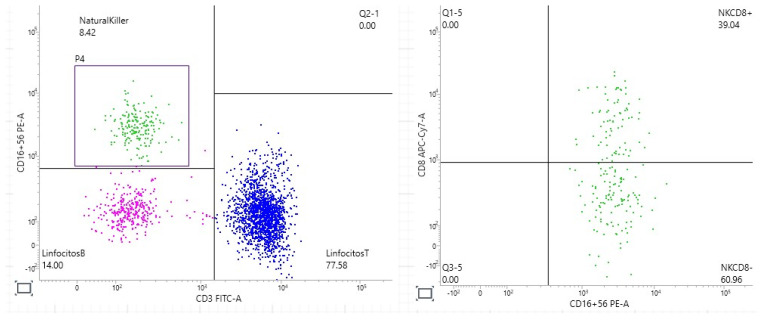
Flow cytometry dot plots that show absence of clonal expansion of NK cells expressing or not CD8+.

The patient did not agree to complete the hematological study (bone marrow aspiration and large granular cell leukemia study). After multiple attempts to reach a consensus on diagnostic test and treatment without success, she was admitted due to fever, nosebleeds and anemic syndrome related to severe thrombopenia plus pancytopenia. Upon admission, she had fever without infection. After multiple blood urine cultures and specific serology, her laboratory tests showed Hb 7.9 g/dL, hematocrit 26.4%, total leucocytes 3770× mm^3^, total neutrophils 1400× mm^3^, platelets 28,000× mm^3^, serum IgA 1004 mg/dL, serum IgE 107 UI/mL, serum IgG 6038 mg/dL, serum IgM 611 mg/dL, ESR 125 mm/hora, CRP 22.4 mg/L, serum amyloid 3.34 mg/L, procalcitonin 0.32 ng/mL, RF 5623 U/L and ACPA > 340 U/mL. Multiple myeloma (absence of serum monoclonal protein, heavy chain expression by immunofixation, urinary monoclonal protein) and large granular lymphocyte leukemia (absence of CD8+ cell proliferation by flux cytometry) were ruled out (Figure 4).

She accepted treatment with biannual rituximab. Until the last review, she had received two cycles of 1000 mg IV (day 0 and day 15), with the last one in March 2024, without complications. There was evidence of improvement in inflammatory activity DAS28PCR, 1.8, anemia and thrombocytopenia, but so far, not for the remaining laboratory parameters. She had Hb 14.5 g/dL, total leukocytes 2000× mm^3^, total neutrophils 1060× mm^3^, platelets 53,000× mm^3^, ESR 121 mm/h, CRP 1.3 mg/L, RF 4935 UI/mL and ACPA > 340 U/mL. She had had mild upper respiratory tract infections managed on an outpatient basis and agreed to continue the treatment but not with aggressive diagnostic tests.

## 4. Discussion

In the 21st century, neutropenia in RA patients is a common finding, with values between 0.5% to 37% (1–3). In our sample of RA patients, it was 18.5% (14.0–25.6%), in agreement with these values. With the introduction of the T2T strategy since 2010 [11], the characteristics and consequences of neutropenia in RA patients have changed. Recent studies report that in early RA without DMARD treatment, neutropenia occurs uncommonly (in less than 1.5% of cases), and congenital neutropenia should be ruled out [1,2,3,4]. In established RA, the prevalence is much higher, and the main cause of neutropenia is treatment with DMARDs, mainly bDMARDs with prevalences of 14.3% to 19%, followed by RA activity. Our retrospective series included patients with a mean of 61 years, long-lasting disease and a high proportion of RF and ACPA positivity, and the frequency and causes of neutropenia were like those in other studies [1,2,3]. Between the biologics, those most common related with neutropenia were IL6i drugs, mainly tocilizumab followed by rituximab, and lastly, by TNFi drugs. scDMARDs were the second most common cause of neutropenia after bDMARDs, mainly methotrexate. And JAKi were the third group related to neutropenia [1,2,3]. These findings were very similar to ours, with 79% of episodes of neutropenia for bDMARDs mainly involving IL6i (59% tocilizumab and 9% sarilumab), followed by TNFi drugs in 24% (18% etanercept, 9% adalimumab and 4% infliximab), and JAKi in 3%. Maybe rituximab was the last due to the low prevalence of use of rituximab in our cohort. In our series and in the recent literature, no cases were related to NSAIDs, maybe because the prevalence of use of NSAIDs has reduced, according to recommendations for RA treatment [12]. The use of oral or parenteral corticosteroids has not been correlated with neutropenia either in our or in other studies [1].

In multivariate analysis, neutropenia was correlated to drugs, especially bDMARDs, particularly IL6i, and with sex and the presence of other cytopenia [1,2,3,10,13]. As with the prevalence of neutropenia, the causes of it are different between DMARDs classes, and even within the same class [1,2,3]. In the case of tocilizumab, moderate (grade III) neutropenia occurred in 7% of RA patients [13]. Although the mechanism of neutropenia associated with tocilizumab is unknown, up-to-date blocking of IL-6 in has an effect on neutrophil recruitment from bone marrow and in the regulation of its selection and expression, but the proposed mechanisms have no effects on the neutrophils function [13,14]. These data are like sarilumab, the other IL6i used in RA patients [15]. For the case of TNFi, the causes of neutropenia are different and varied: decreased upregulation of pro inflammatory cytokines (IL-1, IL-6, IL8 and granulocyte-macrophage colony-stimulating factor), all of them involved in the differentiation and maturation of neutrophils and hematopoietic cells. Also in increases in the peripherical consumption of neutrophils, and imbalances between apoptosis and their survival, mainly accelerated neutrophil apoptosis. Even these effects are different between monoclonal antibodies or soluble receptors against TNF [16,17]. In the case of rituximab, the exact frequency of neutropenia is difficult to estimate due to the highly variable posology in RA patients [1,2,3]. The onset of neutropenia in rituximab patients is late, after 4 weeks of the last dose, without another identifiable cause, and its frequency is about 1.3% to 6.5% [18,19]. Several mechanisms are postulated, highlighting the interference with the output of neutrophils from the bone marrow during B cell recovering, imbalance between granulopoiesis vs. lymphopoiesis, and arrest in the maturation of promyelocyte stage [18,19].

For csDMARDs the prevalence of neutropenia is about 8%, similar between methotrexate, leflunomide or sulfasalazine. Here, the mechanisms are different [1,2,3]. Methotrexate was discovered in the mid-20th century and introduced for the treatment of RA between 1951 and the 1970s; after more than 60 years of use, it is the anchor drug treating RA [20]. With the actual dose regimen of a low dose of methotrexate recommended (between 10 and 25 mg/week plus folic acid supplementation of 5 to 15 mg/week), the frequency of neutropenia is about 6.5% [21]. Data confirmed in other cohorts methotrexate-exposed populations [22]. The cause of methotrexate-related neutropenia is not elucidated but may be related to the competitive inhibition of the enzyme dihydrofolate reductase and depleted hepatic folate stores, without effect in the adenosine pathway [20,21,22]. Whatever the mechanisms of neutropenia associated with DMARDs, in ours and in most of the published works, neutropenia was mild, acute, of a single episode, and was resolved with DMARD dose reductions. As in other series, risk factors of neutropenia were female sex, pre-existing neutropenia, and presence of other cytopenia [1,2,3,10,13,14,15,16,17,18,19,20,21,22]. A specific protocol of cytopenia related to methotrexate [20], IL6i [10,23] and other DMARDs has been developed and should be implemented in clinical practice according to the label. It is simple and includes baseline medical history with emphasis in infection, neutropenia and other medication history; complete blood counts should be taken at 2 to 3 months depending on the clinical condition and type of DMARDs. In addition, vaccination, specific prophylaxis protocols, reduction in the use of steroids, and the management of comorbidities and concomitant drugs related to neutropenia and infection risk (diabetes, lung disease, chronic kidney disease, etc.) should be undertaken. If infection is suspected, early treatment should be initiated to reduce morbidity and mortality [24,25]. In a case of severe neutropenia, either febrile or not, the patient should be hospitalized, and a multidisciplinary team should start the specific protocol [26,27].

Autoimmune causes of neutropenia and Felty syndrome after the introduction of the T2T strategy of RA treatment as cause of neutropenia are rare, as in our series [1,2,3,4,6]. However, in our series, the patients with neutropenia were older and had long-lasting disease. Felty syndrome prevalence was 0,3% of the total. Case 3 showed, in addition, poor therapeutic adherence. The diagnosis of RA activity as cause of neutropenia in Felty syndrome is determined by exclusion, after discarding drug toxicity (DMARDs and non-steroid anti-inflammatory drugs (NSAIDs), and painkillers as the most common), followed by infections. Again, baseline medical history with emphasis on RA activity index, physical examination, folic, B12 or cupper deficiencies should be tested. Acute-phase reactants are usually elevated, except in patients treated with IL6i, where they may be decreased [23]. In some cases, if it is possible, antibodies against granulocytes of pan-RF-γIIIB type can be useful [2]. Once toxicity drugs or other toxic materials, infection or neoplasia have been ruled out, RA treatment should be intensified following the T2T strategy [11,12,20,24,25].

Nowadays, the frequency of Felty syndrome is very low at about 0.5% [6]. In these patients, it is relevant to confirm RA treatment adherence. RA activity searches should be completed with image studies to confirm splenomegaly. Of particular interest is ruling out large granular lymphocyte leukemia and multiple myeloma, as well as some uncommon chronic parasitic and fungal infections. In the multidisciplinary team, the inclusion of a hematologist and infectious disease expert is mandatory [2,3,4,5,6]. Some cases of Felty syndrome have been informed by effective treatment with rituximab, tocilizumab, etanercept or abatacept, even intravenous gamma globulins, without consensus about the best one [28,29,30,31,32,33]. In any case, the intensification of the T2T strategy in consensus with the patient should be implemented.

The study limitations are of course the retrospective design and the absence of a control group. But the results, according to recent previous studies and the scarcity of works with clear prevalence estimations and clinical course of the patients, reinforce the data.

## 5. Conclusions

In the 21st century, neutropenia associated with RA is common and especially related to T2T strategy and DMARDs. Most of the episodes were mild, of short duration and not related to infections. The frequency of classical Felty syndrome is half that of the last century, with effective therapeutic alternatives. 

## Figures and Tables

**Table 1:** Clinical characteristics of patients with neutropenia.

Variable	Neutropenia		Without Neutropenia		*p* Value
	n	%	n	%	
	50	19	220	81	
Female sex	45	90	168	76	0.03
RF+	43	86	184	84	0.6
ACPA+	36	73	175	80	0.3
ACPA+ and RF+	32	65	163	74	0.06
Other cytopenia	21	48	57	27	0.006
Anemia	19	38	47	21	0.01
Thrombocytopenia	11	22	12	5	0.0001
Lymphopenia	2	5	3	1	0.1
Splenomegaly	1	2	0	0	
Felty syndrome	1	2	0	0	
Deaths	2	4	2	1	0.1
DMARD	48	96	205	94	0.5
scDMARD	27	54	140	64	0.1
One	26	52	136	62	0.4
Two	1	2	4	2	0.1
MTX	19	68	102	73	0.1
bDMARD	35	71	128	58	0.08
Monotherapy	1	2	20	7	0.09
Combo with scDMARD	16	32	73	33	0.09
sdDMARD	1	2	20	9	0.09
Combo with scDMARD	0	0	6	3	0.2
	Median	p25–p75	Median	p25–p75	
Age at diagnosis, years	45.7	38.2–53.7	48.5	39.1–57.9	0.1
Age (at time of neutropenia or cut off), years	61.6	49.8–69.2	61.4	53.0–69.5	0.2
Disease duration, (at time of neutropenia or cut off) years	11.6	6.4–21.3	10.4	6.6–17.7	0.5
RF title, U/mL	274	96.2–591.2	236	119.2–367.7	0.2
ACPA title, U/mL	299	87–343.5	340	113.7–359	0.2

RF: rheumatoid factor, ACPA: anti-citrullinated protein antibody positivity, DMARD: disease-modifying anti-rheumatic drugs, scDMARD: synthetic conventional disease-modifying anti rheumatic drugs, MTX: methotrexate, Combo: in combination with, sdDMARD: synthetic directed disease-modifying anti-rheumatic drugs. Data are presented as median and percentiles 25 and 75 (p25 and p75).

**Table 2:** Logistic regression model.

Variable	Odds Ratio	95% Confidence Interval	n = 256 *p* = 0.0001 LR Chi^2^ 42.5 *p* Value
IL6 inhibitors	10.73	3.56–32.2	0.0001
Other cytopenia *	3.83	1.93–7.60	0.0001
Gender, female	5.10	1.36–19.18	0.01
TNF inhibitors	2.50	1.0–6.29	0.05
Rituximab	4.18	0.32–62.8	0.2
DAS28	1.35	0.95–1.90	0.08
Age	0.99	0.95–1.02	0.6
Disease duration	1.01	0.97–1.05	0.5

* includes anemia, thrombocytopenia or lymphopenia. LR Likelihood ratio. DAS28 Disease activity score with 28 joint count.

**Table 3:** Main characteristics of neutropenia episodes.

Variable	n	%
Patients	50	18.5
Number of episodes of neutropenia		
1	26	53
2	7	14
3	9	18
4	5	10
5	1	2
7	1	2
Total	99	100
Duration of neutropenia		
Acute	58	59
Chronic	41	41
Severity of neutropenia		
Mild	73	74
Moderate	20	20
Severe	6	6
Cause of neutropenia		
Drugs	78	78
RA activity	13	13
Systemic viral infections	3	3
Hematologic cancer	1	1

**Table 4:** Drugs related to neutropenia episodes.

Synthetic Conventional DMARDs Either in Monotherapy or in Combination with Biologic DMARDs				
Type	Monotherapy	Combo with Synthetic Conventional DMARDs	Combo with Biologic DMARDs	Total
Total				
	n (%)	n (%)	n (%)	n (%)
Methotrexate	9 (20)	1 (2)	18 (41)	28 (64)
Leflunomide	3 (7)	1 (2)	6 (14)	10 (23)
Hydroxychloroquine	0	1 (2)	5 (11)	6 (14)
Total	12 (27)	3 (7)	29 (66)	44 (100)
Biologic DMARDs related to neutropenia				
Tocilizumab	32 (40)		15 (19)	47 (59)
Etanercept	9 (11)		5 (6)	14 (18)
Adalimumab	3 (4)		4 (5)	7 (9)
Sarilumab	5 (6)		2 (2)	7 (9)
Infliximab	0		3 (4)	3 (4)
Rituximab	1 (1)		0	1 (1)
Total	49 (62)		29 (37)	79 (100)
Targeted synthetic DMARDs related to neutropenia				
Baricitinib	2 (66)		0	2 (66)
Upadicitinib	1 (33)		0	1 (33)
Total	3 (-)		0	3 (100)
Other treatments				
Rucaparib	1 (50)		0	1 (50
Carboplatin/ paclitaxel	1 (50)		0	1 (50)
Total	2 (100)		0	2 (100)

DMARDs: disease-modifying anti rheumatic drugs.

## Data Availability

Data have been stored in our research archives according to local legislation.
